# Evaluation of Red Palm Weevils (*Rhynchophorus ferrugineus*: Curculionidae) for Putative Oxidation of Ingested Polystyrene and Polyurethane and Their Gut Microbiota Response

**DOI:** 10.3390/insects16060587

**Published:** 2025-06-02

**Authors:** Khanchai Danmek, Pichet Praphawilai, Sampat Ghosh, Chuleui Jung, Saeed Mohamadzade Namin, Phattharawadee Aedtem, Bajaree Chuttong

**Affiliations:** 1 Biotechnology Program, School of Agriculture and Natural Resources, University of Phayao, Phayao 56000, Thailand; khanchai.da@up.ac.th; 2 Office of Research Administration, Chiang Mai University, Chiang Mai 50200, Thailand; pichet.p@cmu.ac.th; 3 Meliponini and Apini Research Laboratory, Department of Entomology and Plant Pathology, Faculty of Agriculture, Chiang Mai University, Chiang Mai 50200, Thailand; 4 Agricultural Research Institute, Gyeongkuk National University, Andong 36729, Republic of Korea; sampatghosh.bee@gmail.com; 5 Department of Life Science, Sardar Patel University, Balaghat 481331, Madhya Pradesh, India; 6 Department of Plant Medicals, Gyeongkuk National University, Andong 36729, Republic of Korea; 7 Department of Horticulture, College of Agricultural Science, Oregon State University, Corvallis, OR 97331, USA; saeedmn2005@gmail.com; 8 Department of Halal Culinary and Nutrition, Faculty of Science Technology and Agriculture, Yala Rajabhat University, Yala 95000, Thailand; phattharawadee.a@yru.ac.th

**Keywords:** red palm weevil, plastic ingestion, polystyrene, polyurethane, microbiome

## Abstract

This study assessed the growth performance of red palm weevil (RPW) (*Rhynchophorus ferrugineus*: Curculionidae) larvae on a liquid diet of yeast-enriched potato dextrose broth (control) and on diets with added polystyrene and polyurethane. For 15 days of diet exposure, the growth and survival, plastic degradation, and gut microbiota of larvae were examined. RPWs showed higher survival rates under polystyrene and polyurethane treatments than in the control group. Head diameter showed a higher trend under polyurethane treatment than under the other treatments. Treated plastics were partly degraded after a 15-day exposure. Further analysis of plastic residues from frass revealed significant differences in Fourier Transform Infrared Spectroscopy (FTIR), with decreased intensity of characteristic peaks compared to frass from larvae fed in the control. Gut bacterial communities in the gut of RPW larvae showed that plastic feeding did not significantly alter the presence of key microbial taxa, but members of *Firmicutes* and *Proteobacteria* were higher in the plastic treatment, showing preliminary signs of plastic oxidation and degradation. Overall, these findings provide evidence that ingestion of PS and PU by RPW larvae supports their survival and alters their gut microbiota, possibly due to plastic degradation, paving the way for further research into the interactions between RPWs, their microbiome, and key functional activities, with implications for plastic waste management and recycling.

## Simple Summary

The accumulation of global plastic waste poses a significant environmental challenge, necessitating the development of sustainable plastic waste management methods. Exploration of the use of insects, such as mealworms and wax moth larvae, to expedite the degradation of plastic materials is unceasing. This research is the first investigation of the potential of red palm weevil (RPW) larvae for managing polystyrene and polyurethane plastic waste. These findings represent evidence that RPW larvae possess the capacity to degrade plastic materials, and open up a novel avenue for studying the interaction between RPW and associated gut microbiota and their key activities, with significant implications for the management and recycling of plastic waste.

## 1. Introduction

In 2015, global plastic waste accumulation was estimated to reach 5.8 billion tons, with polypropylene and acrylic fibers accounting for approximately 700 million tons [1], and global plastic production continued to rise to 400.3 million tons in 2022 and 413.8 million tons in 2023 [2,3]. These materials are widely used across various industries, including the furniture, automotive, and construction industries [4,5]. Data highlight the urgent need for the development of sustainable plastic waste management methods, as the chemical structure of these plastics makes them resistant to natural degradation, leading to the accumulation of plastic waste in landfills [6,7]. Consequently, finding effective disposal or recycling methods presents a significant challenge in mitigating the environmental impact of plastic waste [8]. Interestingly, recent studies have reported the potential of certain insect species in degrading synthetic polymers, primarily facilitated by their gut microbiota, offering a promising, biologically driven approach to plastic bioremediation [9,10,11]; however, to the best of our knowledge, this is the first report demonstrating such a potential of red palm weevils (RPWs).

The use of insects capable of degrading plastic materials has emerged as a promising solution for applying environmentally friendly biological processes [9,10,11]. The biodegradation of plastics by insects is expected to play a role in mitigating plastic waste, providing a sustainable solution to the growing global plastic waste crisis. Prior to this study, several studies have explored the use of insects in plastic degradation from various perspectives. For instance, mealworm larvae (*Tenebrio molitor* L.) have been observed to consume and partially degrade polyurethane plastic, albeit at a slower rate compared to polystyrene (PS). This degradation process is hypothesized to be facilitated by enzymatic mechanisms akin to those observed in the degradation of PS, potentially involving a combination of gut microbiota and the digestive enzymes of insects [10,12,13]. Similarly, research by Helmberger et al. [14] examined the plastic degradation capacity of various insects, including cricket (*Gryllodes sigillatus* Walker), isopod (*Oniscus asellus* L.), and superworm beetle larvae (*Zophobas morio* Fabricius). Recently, Peng et al. demonstrated that *Zophobas atratus (conspecific with Zophobas morio and Zophobas rugipes)* larvae from sources in China and the U.S.A. were capable of degrading PS and low-density polyethylene (LDPE) [15]. These studies found that these insects consume plastic and excrete it as frass, potentially contributing to the formation of microplastics in environments contaminated with plastic waste. A similar capacity of darkling beetle larvae (*Plesiophthalmus davidis*) to degrade PS was shown [10,11]. Nearly all major plastics, including polyethylene (PE), polypropylene (PP), polyvinyl chloride (PVC), polyethylene terephthalate (PET), polyurethane (PUR), and PS, have been shown to undergo biodegradation by *T. molitor* and ten other insect species from the Tenebrionidae and Pyralidae families. This biodegradation occurs through symbiotic interactions or the combined efforts of the host and its gut microbes, facilitating the rapid depolymerization and breakdown of plastics, often with half-lives measured in hours [16]. However, this ability of some insects may lost when the gut microbiota are inhibited by antibiotic treatment [17].

Red palm weevil (RPW) larvae (*Rhynchophorus ferrugineus*: Curculionidae), a major pest of palm trees and an edible insect in many Southeast Asian regions, have gained recent attention not only for their ecological and agricultural relevance, but also for their unique gut microbiome. Recent research by Elkraly et al. [18] has highlighted the diverse functional roles of the RPW gut microbiota, including its potential involvement in pesticide detoxification, thermal stress tolerance, and possibly nutrient digestion, all of which point to the adaptive complexity of the RPW gut ecosystem. These functions are largely attributed to the activity of gut-associated microbial communities, suggesting a highly versatile metabolic potential. Therefore, it is plausible that RPW larvae may also possess plastic-degrading capabilities, facilitated by the synergistic action of their digestive enzymes and microbial partners. Based on our initial observation of larvae chewing through plastic materials and previous reports of plastic degradation by other beetle larvae, such as *T. molitor*, we hypothesize that red palm weevil larvae can actively degrade plastic, offering a potential bioremediation strategy for plastic waste management, presumably primarily through the activity of their gut microbiota.

Plastic pollution is a major environmental concern, especially in Southeast Asia. Countries like Thailand, which produce large quantities of plastic waste, also serve as global hubs for RPW farming [19]. Exploring biological solutions for plastic degradation is therefore of great importance, and recent research suggests that certain insects, including the RPW, may have the potential to contribute to this effort.

This study indicates that RPWs can ingest plastic, such as PS and PU, raising the possibility that these insects could play a role in bioremediation. However, understanding the implications of plastic ingestion is crucial. In particular, analyzing the larvae’s gut microbiome can provide valuable insights into the mechanisms of plastic degradation, such as identifying microbial taxa involved and understanding how microplastics may accumulate within the insect bodies. This is important because the formation and release of microplastics through insect excretion could pose risks to human health if larvae are consumed, as well as contributing to environmental concerns surrounding the release of microplastics into ecosystems [9,13,20].

Based on the findings outlined above, we hypothesize that RPW larvae may possess a unique ability to degrade plastics through their digestive system. To investigate this, our study focuses on assessing the larvae’s growth performance and digestive capacity when fed PS and PU. Additionally, we analyze changes in their gut microbiota and utilize Fourier Transform Infrared Spectroscopy (FTIR) to evaluate structural modifications of the plastics after ingestion. By examining the interaction between plastic consumption, gut microbiota dynamics, and plastic degradation, this research aims to evaluate the potential of RPW larvae as a sustainable biological agent for plastic waste management. Ultimately, our findings could contribute to understanding both the biodegradation process and the ecological and health implications associated with microplastics generated by insect digestion.

## 2. Materials and Methods

### 2.1. Sources of Red Palm Weevil (RPW) Larvae and Plastics

In this study, second-instar red palm weevil (RPW) larvae, approximately 20 days old, were obtained from a farmer in the Mae Tang district, Chiang Mai Province, Thailand (19°03′08″ N, 98°57′00″ E). The larvae were identified as *R. ferrugineus* based on morphological characteristics. At this developmental stage, the larvae exhibited a head-to-mouth size of approximately 4 mm and a weight ranging from 3.5 to 4.0 g. The larvae displayed high consumption rates and demonstrated strong adaptability for rearing in plastic containers.

Two types of plastic, specifically, PS and PU, were utilized to investigate the feeding preferences of the RPW larvae. Both plastic samples, used as feedstock for the larvae, were procured from supermarkets in Chiang Mai Province. The plastic samples were cut into standardized rectangular dimensions (length × width × height = 2 × 1 × 1 cm), soaked in 70% alcohol for 30 s, and subsequently rinsed three times with sterilized water to remove surface contaminants. The plastic blocks were then dried at 30 °C under sterile conditions and stored in a desiccator until required for experimental use.

### 2.2. Diet Preparation

The nutritional formula used in this study for the RPW larvae was based on the previous research of Promwee et al. [19], who investigated the appropriate nutritional diet for red palm weevils (RPWs) and concluded that their medium should contain at least 5% protein. To prepare the diet treatment, 120 g of yeast extract was added to 800 mL of potato dextrose broth (PDB), with the final volume adjusted to 1000 mL at a pH of 6.0. This adjustment ensured that the final yeast-enriched PDB (YPDB) solution contained at least 5.0% protein, which is an optimal diet for RPW growth and was used as a control in this experiment. The clear-liquid nature of the YPDB medium provided significant advantages as a control, allowing for easy observation and sample collection. Other treatments were either 100 pieces of PU plastic (polystyrene group) or 100 pieces of polyurethane plastic (polyurethane group) added to YPDB. The transparency of the medium enabled efficient collection of RPW excrement and plastic particles suspended in the liquid, facilitating accurate analysis. Manual separation was performed using tweezers and fine sieves to minimize contamination of the samples. For smaller fragments indistinguishable by visual inspection, further analysis, such as FTIR, was performed to characterize the remaining plastics.

### 2.3. Growth Performance and Collection of Samples

Thirty larvae were housed each in three replicate containers per experimental group (control, polystyrene, and polyurethane). All containers were maintained under controlled environmental conditions of 30 ± 1.0 °C and 60 ± 1.0% relative humidity in complete darkness. After the larvae were incubated, the numbers of living and dead larvae were counted to determine the survival rate over the incubation period. Dead larvae were promptly removed upon detection to prevent contamination. To ensure adequate nutrition and prevent feed shortages, 40 mL of fresh YPDB was added every 3 days. Growth performance was evaluated using 10 larvae from each group. After 15 days of rearing, the body weight (g) of the larvae was measured. The study evaluated the growth performance of RPW larvae using three different treatments: no plastic, PS plastic, and polyurethane plastic. Key parameters measured included head diameter (mm), live weight (g), and survival rate (%). These parameters were recorded at three time points: 20 days of age (prior to treatment exposure as a baseline), 7 days after exposure (27 days of age), and 15 days after exposure (35 days of age). This approach allowed us to monitor the effects of different plastic treatments on larval growth and survival over time.

### 2.4. Collection and Characterization of RPW, Residual Plastics, and Frass

The samples were collected after 15 days of rearing, ensuring that all larvae from each treatment group were of the same age. For gut microbiome analysis, five larvae from each treatment group were randomly selected and preserved in 95% ethanol (analytical-grade) for further analysis. These samples were stored at −80 °C to maintain their integrity. The gut samples were not pooled together; each sample was collected individually to preserve the specific microbial diversity associated with each treatment group. Additionally, residual plastics and frass from each experimental group were collected separately and stored at −20 °C for further characterization.

### 2.5. Fourier Transform Infrared Spectroscopy (FT-IR) Analysis

The macrostructures of the plastic samples before and after rearing, as well as those within the midgut of RPW larvae, were examined using an Olympus BX53 Digital Upright Microscope (Olympus Corporation, Tokyo, Japan). Photographs were captured at 40× magnification using an IMTcamCCD5 PLUS camera (IMT i-Solution, Inc., Vancouver, BC, Canada) for imaging and scaling. These samples were collected at 15 days of incubation, which was the same age used for all experimental groups, starting from the initiation of the experiment. Additional characterization of the residual polymer was conducted through Fourier Transform Infrared Spectroscopy (FT-IR) using an EQUINOX 55 FT-IR Spectrometer (Bruker Corporation, Ettlingen, Germany). The FT-IR analysis covered the wavenumber range of 500–4000 cm^−1^ to identify the functional groups present in the residual PS and PU plastic.

### 2.6. Diversity Gut Microbiome

At the end of the experiment, 15 days after the initiation of the rearing process, RPW larvae were individually surface-sterilized with a 90% ethanol solution for 30 s, followed by washing in sterilized distilled water. The entire gut was dissected from five larvae per treatment group using sterile scissors and forceps, facilitated by gently pulling on the sting shaft [21,22,23]. DNA extraction was performed using the QIAamp DNA Mini Kit (QIAGEN, Hilden, Germany), and the quantity and quality of the DNA were assessed with a Micro UV-Vis Spectrophotometer (Life Sciences, Zhengzhou, China). DNA purification was carried out using the OneStep PCR Inhibitor Removal Kit. The extracted DNA samples were stored at −20 °C prior to library preparation [24].

The V4 region of the 16S rRNA gene was PCR-amplified, using the primers 515F (5′-GTGCCAGCMGCCGCGGTAA-3′) and 806R (5′-GGACTACHVGGGTWTCTAAT-3′), with a Thermal Cycler (Bioer—GeneExplorer, Hangzhou, China). Sequencing was conducted using the Illumina MiSeq platform (Macrogen, Seoul, Republic of Korea). A metagenome amplicon sequencing approach was employed, with paired-end reads of 301 bp length in FASTQ format. Sequence analysis was performed using the Qiime2 platform, employing the DADA2 algorithm to calculate alpha diversity. Taxonomic annotation was conducted using the SILVA v1382 database [24].

### 2.7. Statistical Analysis

Data that followed a normal distribution were analyzed using one-way analysis of variance (ANOVA), followed by a post hoc Tukey’s b test. A significance level of *p* < 0.05 was applied for all statistical tests, and the analysis was conducted using SPSS version 26.0 (IBM Co., Armonk, NY, USA). The statistical significance of differences in time distributions between groups was also assessed.

## 3. Results

### 3.1. Growth Performance and Survival Rate of RPW Larvae Reared on Plastic-Containing Diets

**Figure 1:**
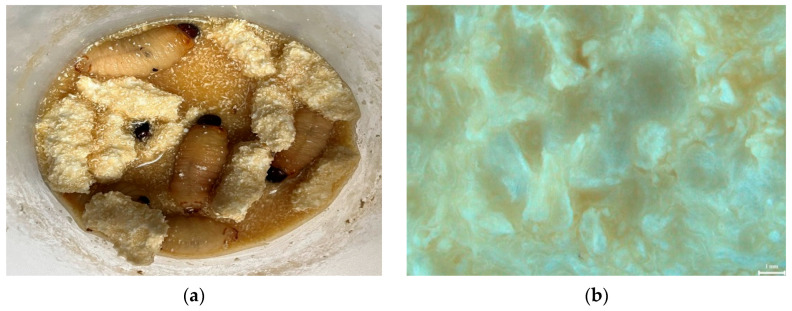

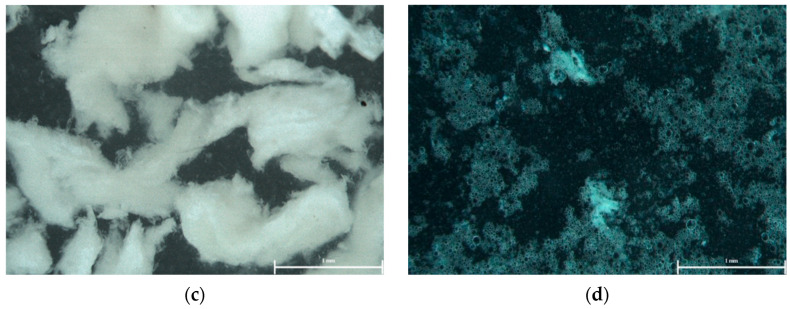
Appearance of polystyrene treatment in YPDB with RPW larvae after 15 days of exposure: (**a**) polystyrene pieces and RPW larvae within liquid diet of YPDB, (**b**) surface structure of plastic block, (**c**) residual plastic in the liquid, and (**d**) plastic particles in gut of larvae.

**Figure 2:**
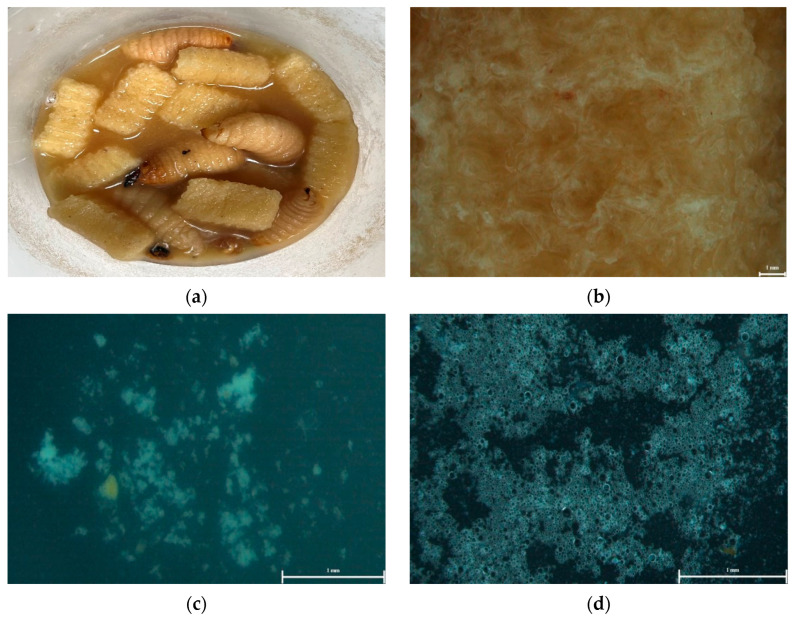
Appearance of polyurethane treatment in YPDB with RPW larvae after 15 days of exposure: (**a**) polyurethane pieces and RPW larvae within liquid diet of YPDB, (**b**) surface structure of plastic block, (**c**) residual plastic in the liquid, and (**d**) plastic particles in gut of larvae.

The growth performance of RPW larvae showed that head size increased as the larvae aged (Table 1). Larvae at 27 and 35 days had a significantly larger head diameter compared to those at 20 days, the starting point of the experiment (*p* < 0.05). Even without statistical significance (*p* > 0.05), the head capsule width of the polyurethane group was higher than that of the other groups (Table 1). The live weight of larvae showed a trend of decrease as the larvae aged, but was seemingly heavier in the PU and PS groups than in the control group (*p* = 0.066). Contrary to expectations, the larvae in the PS and PU groups exhibited a 100% survival rate, whereas the control group showed a significantly lower survival rate of 41.1% (*p* < 0.05). These findings suggest that the consumption of plastic had no impact on survival. The RPW larvae consumed the plastic, as evidenced by fragments found in their digestive systems (Figure 1 and Figure 2). The larvae mechanically chewed the plastic into smaller pieces, increasing its surface area and potentially enhancing enzyme activity to change the polymer structure. However, fragments smaller than 5 mm were still observed in larvae fed both plastic types, indicating incomplete breakdown. PS consumption was more noticeable due to its opaque white color, while transparent polyurethane fragments were harder to detect, blending with the medium and waste.

### 3.2. Plastic Transfomation by RPW Larvae

**Figure 3:**
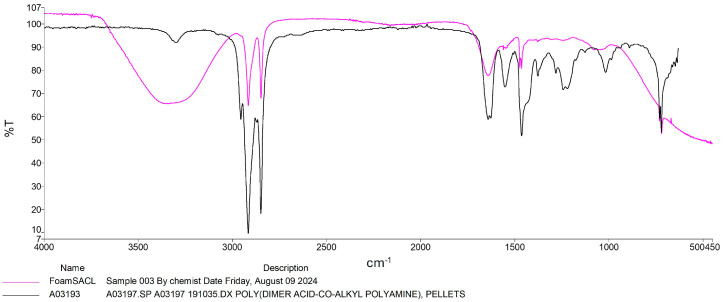
FTIR spectra of polystyrene polymer (black line) and residual polystyrene polymer (violet line) by RPW larvae.

While some modifications in plastic structure were detected, these observations are only preliminary, and do not constitute definitive evidence of biodegradation. FTIR spectra of residual plastics from larvae fed PS and PU showed decreases in characteristic peaks. The FTIR spectra showed clear changes in the remaining polymers after the RPW larvae were fed with plastic, compared to the control. Peaks characteristic of PS plastics (Figure 3) showed a decrease in intensity, particularly in the low-wavenumber region (500–1600 cm^−1^), associated with C-H bending and C-C stretching in aromatic rings, as well as in the high-wavenumber range (2800–3300 cm^−1^), corresponding to C-H stretching. Additionally, the spectra of the remaining polymers displayed peaks related to oxygen, indicating the incorporation of oxygen during the modification process, particularly C-O stretching (1000–1200 cm^−1^) and alcohol group stretching in the 3000–3500 cm^−1^ region. The modification by the RPW larvae of the structure of the PS sample was observed to be more extensive than that of the PU sample. However, the exact mechanism of degradation, whether due to the larvae activity or the enzymes they produce, remains unclear, especially regarding the oxidation of ether bonds in both samples.

**Figure 4:**
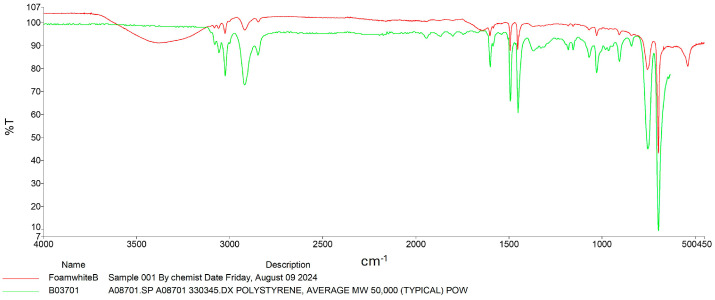
FTIR spectra of polyurethane polymer (green line) and residual polyurethane polymer (pink line) by RPW larvae.

The modifications in PU structure caused by the RPW larvae were also investigated using FT-IR. Similarly to PS, significant reductions were observed in peaks associated with absorption bands around 3200–3400 cm^−1^, which are characteristic of N-H stretching and O-H stretching due to hydrogen bonding [25]. In addition, the absorption peaks around 2780–2950 cm^−1^, present in both samples, indicated C-H stretching. Additionally, in the range of 1000–1700 cm^−1^, both PU without and with RPW larvae showed peaks corresponding to C-O-C stretching (1000–1200 cm^−1^), C=C stretching in benzene rings (1531 cm^−1^), and C=O stretching (1726 cm^−1^) [5,26]. Although the biodegradation mechanism of PU by RPW larvae remains unclear, it is hypothesized to be associated with oxidation reactions. It is still uncertain which enzymes may be involved in the oxidation of PU [5]. Therefore, identifying potential enzymatic activities involved in the deformation of PU, whether originating from RPW larvae or gut microbiota, represents an important area for future research (Figure 4).

### 3.3. Gut Microbiota of RPW Larvae Reared on Plastic-Containing Diets

**Figure 5:**
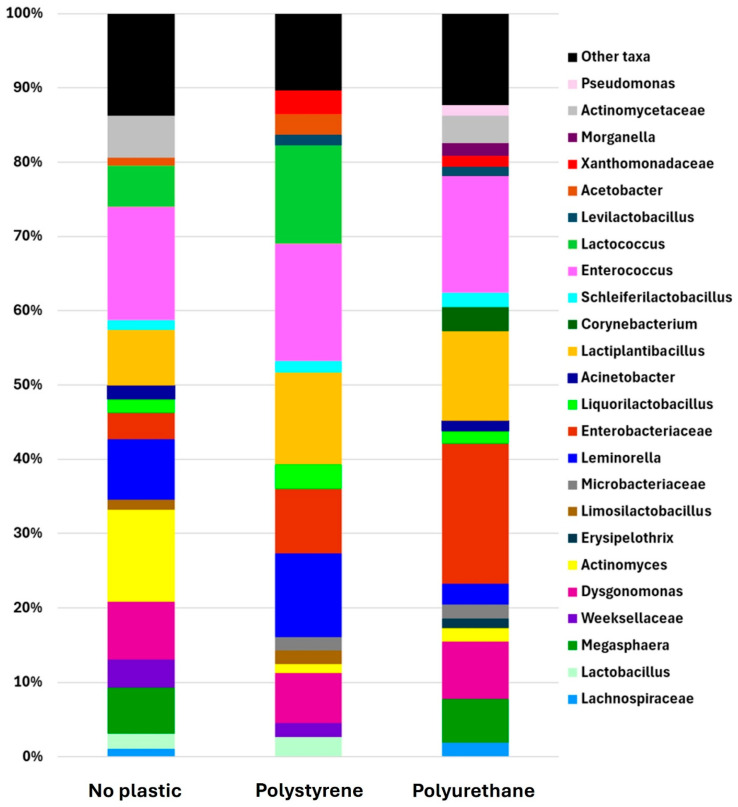
Compositions of gut microbial communities at genus level of RPW larvae among treatments (control, polystyrene, and polyurethane) after 15 days of exposure. Bacterial taxa with relative abundances lower than 1% are present as other taxa.

The gut microbiome analysis of RPW larvae across different dietary treatments revealed the prominent bacterial taxa present in their digestive systems. The major taxa (genus) identified included *Actinomyces*, *Dysgonomonas*, *Enterobacteriaceae*, *Enterococcus*, *Lactiplantibacillus*, *Leminorella*, *Liquorilactobacillus*, and *Schleiferilactobacillus*, which consistently appeared in significant proportions (greater than 0.01) in the gut of RPW larvae across all experimental diets, including those with and without plastic. Among these, *Enterococcus* was the most prevalent genus, comprising approximately 15% of the total bacterial population across all treatment groups, followed by *Dysgonomonas*, accounting for 6.7% to 7.8% of the total bacterial community (Figure 5). Both *Liquorilactobacillus* and *Schleiferilactobacillus* exhibited stability across both plastic and non-plastic conditions, suggesting their consistent presence across varying environmental conditions. Among the commonly detected bacterial groups, *Enterobacteriaceae* showed distinct distribution patterns depending on the plastic type, with relative abundances of 3.5% in the no-plastic group, 8.6% in the PS group, and 18.8% in the polyurethane group. Similarly, the relative abundance of *Lactiplantibacillus* increased from 7.4% in the no-plastic group to 12% in both the PS and PU groups. In contrast, *Actinomyces* exhibited a significant decline, decreasing from 12.3% in the no-plastic group to 1.2% and 1.8% in the PS and polyurethane groups, respectively.

**Figure 6:**
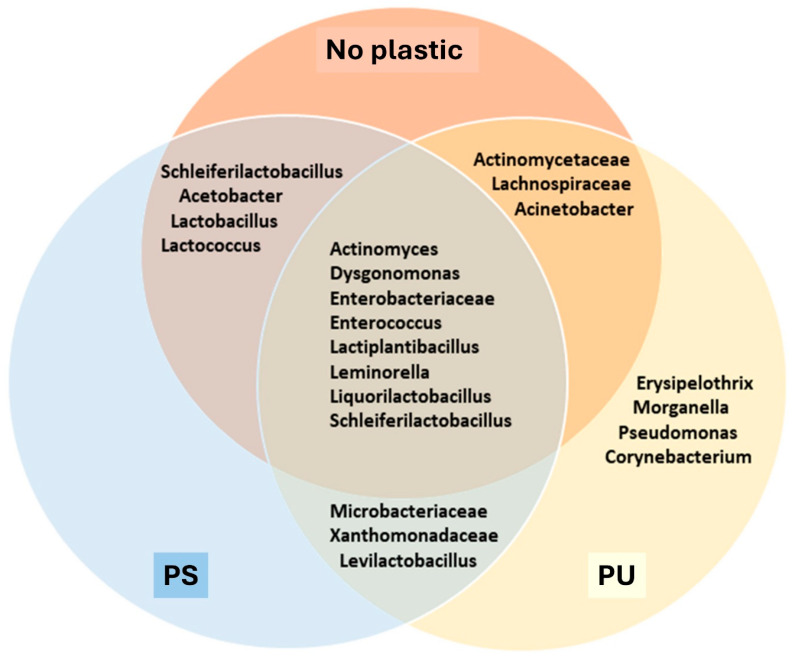
Venn diagram showing shared and exclusive major bacterial taxa in digestive systems of each treatment group (control, PS = polystyrene and PU = polyurethane).

The analysis identified several bacterial taxa (family) shared between the no-plastic and polyurethane groups, including *Actinomycetaceae*, *Lachnospiraceae*, and *Acinetobacter.* In contrast, Acetobacter, Lactobacillus, and Lactococcus were common to both the no-plastic and PS groups (Figure 5). *Lactococcus* showed a significant increase in abundance, rising from 5.6% in the no-plastic group to over 13% in the PS group. Among the major bacterial taxa identified, *Microbacteriaceae*, *Xanthomonadaceae*, and *Levilactobacillus* were shared between the PS and polyurethane groups, but appeared as minor taxa in the no-plastic group (Figure 6). Additionally, the four taxa *Erysipelothrix*, *Morganella*, *Pseudomonas*, and *Corynebacterium* emerged as dominant taxa in the polyurethane group. Moreover, *Pseudomonas* was exclusively present in the polyurethane group, whereas *Erysipelothrix* was absent in the no-plastic control.

**Figure 7:**
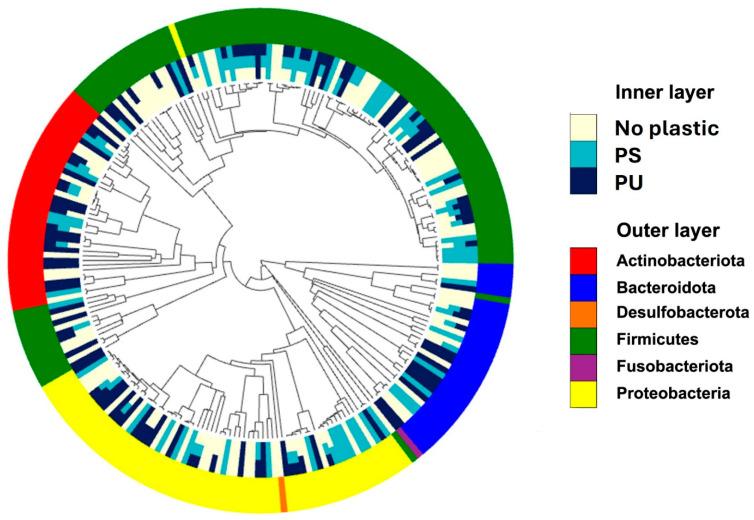
The phylogenetic tree illustrates the bacterial community composition in the gut microbiome of RPW larvae under three different treatments: no plastic, polystyrene (PS), and polyurethane (PU). The central tree shows the evolutionary relationships among the bacterial taxa, with each branch representing a specific taxon. Surrounding the tree, the inner ring indicates the relative abundance and distribution of these taxa across the three treatments. The outer ring categorizes the bacterial taxa at the phylum level.

The phylogenetic tree reveals significant differences in the gut microbiome composition of RPW larvae under different treatments (Figure 7). *Firmicutes* and *Proteobacteria* were the most abundant taxa (phylum), especially in the PS and PU groups, indicating their potential role in plastic degradation or adaptation to the altered gut environment. Conversely, taxa such as *Bacteroidota* and *Desulfobacterota* were less prevalent. The no-plastic treatment maintained a more balanced microbial composition, with relatively equal representation across several phyla, compared to the plastic-treated groups, which showed shifts favoring specific phyla like *Firmicutes*. The findings revealed dynamic shifts in the gut microbiota composition of RPW larvae across different treatment groups, particularly in response to plastic-containing diets. Specific bacterial taxa, such as members of *Firmicutes* and *Proteobacteria*, were identified as being associated with plastic degradation. These alterations underscore the adaptability of the gut microbiome in RPW larvae to varying environmental and dietary conditions, highlighting potential microbial contributors to the digestion and breakdown of synthetic materials.

## 4. Discussion

This study provides the first evidence that RPW larvae can ingest and survive on a diet containing PS and PU plastic pieces supplemented with YPDB, demonstrating their capacity for plastic transformation. While no prior studies have examined the effects of these two plastic types on RPW larvae, the findings suggest that their inclusion does not adversely impact larval growth. No mortality was observed in the plastic-fed groups, with a 100% survival rate throughout the experiment, while the control group exhibited significantly higher mortality over time (Table 1), demonstrating the larvae’s tolerance to plastic consumption. The presence of plastic in the diet may create a favorable environment for RPW larvae by preventing drowning, maintaining humidity, and providing a material for chewing. In contrast, the higher mortality observed in the liquid medium without plastic may be attributed to various factors, such as the weevils’ drowning to death or experiencing stress from a lack of chewable material. Analysis of the larvae’s digestive tracts revealed plastic particles smaller than 5 mm, consistent with microplastic accumulation as defined by Barnes et al. [27] and Avio et al. [28]. This suggests that RPW larvae can incorporate plastic-derived microplastics, raising concerns for human health, especially in regions like Southeast Asia, where they are consumed as food, including in Thailand [29].

If RPW larvae consume plastic-contaminated feed, these plastics may transfer to humans through consumption, potentially causing adverse health effects. Microplastics are linked to risks such as inflammation, toxicity, and chemical transfer [30]. While we did not specifically assess the chemical composition of the degraded plastic fragments, it is crucial to investigate whether toxic byproducts are generated during the transformation process. Future studies should incorporate chemical analysis of these byproducts and toxicity assays to assess potential environmental and health risks. Similarly, research by various scientists has demonstrated that certain types of microplastics are more resistant to degradation, while others may degrade over time. The degradation process is influenced by environmental factors, such as the type of insect, conditions that facilitate plastic degradation, and microbial activity within the digestive system [10,11,12,31,32,33]. Therefore, further research is needed to explore the long-term effects of plastic ingestion in RPW larvae, and to determine whether these plastics can degrade within the digestive system over extended periods.

Previous studies on mealworms (*T. molitor*) and superworms (*Z. atratus*) have demonstrated similar degradation capabilities for PS and PU [13,16,34]. The results from the FTIR analysis in this study further support the occurrence of structural changes in the plastics, evidenced by the spectral changes associated with polymer breakdown. The findings align with previous research confirming the biodegradation of these plastic materials by insect larvae. For instance, Peng et al. [20] used FTIR to validate the structural changes in plastic materials, demonstrating the degradation of aromatic ring structures and the incorporation of oxygen atoms, suggesting the breaking down of polymer chains. The observed reduction in the intensity of characteristic peaks further supports the conclusion that RPW larvae can break down these materials. The more significant degradation of PS compared to PU may suggest that RPWs exhibit varying transformation efficiencies for different plastic materials. However, the mechanisms of plastic biodegradation by RPWs remain unclear. FTIR spectral analysis provides evidence of RPWs’ effective deformation capabilities, which aligns with previous studies on other insect species, highlighting the potential of RPWs in environmental plastic waste management [13,20]. The extent of degradation appeared greater for PS than for PU. While these FTIR findings strongly support modification of polymer composition, they do not definitively establish the precise mechanisms (enzymatic or microbial) responsible. These spectral changes suggest some degree of chemical alteration, potentially indicative of initial oxidative processes. However, without further analytical confirmation, we cannot conclusively attribute these changes to active biodegradation. Future studies employing techniques such as enzymatic assays and microbial community analysis will be needed to fully elucidate the mechanisms involved in this process. Typically, the degradation of plastic particles in these insects occurs through oxidation and hydrolysis, wherein polymer bonds are cleaved into smaller, more manageable molecules. These smaller molecules are then metabolized further by microbial symbionts residing within the insect gut, ultimately reducing the plastic particles into biomass and other byproducts [35]. The proposed mechanism indicates that these larvae may utilize plastic as a feed source, thereby accelerating the breakdown and mineralization of ingested plastic as it passes through the intestinal gut [8,36]. The data corroborate previous research that has demonstrated the crucial role of microorganisms in the insect gut in the plastic degradation process. Bacteria such as *Pseudomonas putida*, *Enterobacter asburiae*, and *Bacillus* spp., which are present in the insect digestive system, have demonstrated the ability to degrade polyurethane plastics composed of polyether structures [12]. These findings are significant in developing strategies to utilize insects for plastic waste management, particularly in response to the increasing global volume of plastic waste.

The consistency in the presence of certain bacterial groups, such as *Enterococcus*, *Dysgonomonas*, *Liquorilactobacillus*, and *Schleiferilactobacillus*, across both plastic and non-plastic feeding treatments points to their potential importance in the gut ecology of RPW larvae. *Enterococcus* was found to be the dominant genus, which suggests its significant role in larvae gut health and metabolism. Previous studies have shown that a high abundance of Enterococcus in the gut of *Galleria mellonella* larvae is associated with increased expression of immune response genes, indicating its role in enhancing host defense mechanisms [37]. While its exact function in RPW larvae remains to be fully elucidated, its consistent presence across all dietary conditions suggests it may contribute to survival, growth, and overall gut stability. Further research is needed to determine whether Enterococcus plays a similar immunomodulatory role in RPW larvae or supports plastic digestion through metabolic activity [38,39]. *Dysgonomonas*, a facultative anaerobe known for its fermentative metabolism, was another prominent genus. The stability of its abundance across treatments could indicate its crucial role in maintaining the gut acidic environment (average pH of 3.6–3.7). Its ability to produce acids without gas could provide a competitive advantage in the larvae’s high-sugar, low-oxygen gut environment, as described by Hofstad et al. [40]. Although the specific roles of *Liquorilactobacillus* and *Schleiferilactobacillus* in insects have not been well documented, lactic acid bacteria, in general, are known to contribute to gut homeostasis, carbohydrate metabolism, and fermentation processes in insect hosts [41]. Their presence in RPW larvae across all diets suggests they may play a role in gut microbiota stability, possibly aiding in nutrient digestion or microbial interactions. Future studies should explore their functional significance in RPWs and whether they contribute to the digestion of plastic-derived compounds.

The observed increase in the abundance of *Enterobacteriaceae*, particularly in the polystyrene group, indicates a growing role of this bacterial family in the gut microbiome of RPW larvae when fed plastic, especially polyurethane. A similar trend in the abundance of Enterobacteriaceae has been documented in other insect species, such as *T. molitor* larvae, when provided diets containing polystyrene, polyether, and polyurethane plastics [42]. Furthermore, the observed increase in *Lactiplantibacillus* suggests its involvement in the digestion of plastic-derived materials, reinforcing the hypothesis that these bacterial taxa are specifically adapted to metabolize plastics within the RPW larvae diet. In contrast, the significant decline in the abundance of *Actinomyces* in both plastic-fed groups implies that the presence of plastic may disrupt the equilibrium of the gut microbiome, potentially favoring bacterial taxa that are better suited to processing plastic materials.

The consistent presence of *Actinomycetaceae*, *Lachnospiraceae*, and *Acinetobacter* in the no-plastic group suggests that these bacterial taxa are not exclusively associated with plastic in the diet. Their high abundance in the no-plastic group indicates their general presence in the gut microbiome of RPW larvae, regardless of plastic inclusion. However, the demonstrated increase in *Lactococcus* abundance in the polystyrene group suggests its potential role in polystyrene degradation. These findings are consistent with previous research, which has identified *Lactococcus* as a key bacterium in the breakdown of polystyrene [43,44], further supporting its involvement in the digestion of plastic-derived materials in the diet of RPW larvae.

The findings indicate a potential association between the composition of bacterial communities and the inclusion of plastic in the larval diet. The increased abundance of *Xanthomonadaceae* observed in the polystyrene and polyurethane treatment groups suggests a possible role for this bacterial family in plastic biodegradation, consistent with previous studies identifying members of *Xanthomonadaceae* as contributors to polystyrene degradation [45]. Furthermore, earlier research has established the capacity of *Pseudomonas* and *Erysipelothrix* to degrade polyether-based polyurethane [12,36,45], underscoring their likely involvement in the breakdown of synthetic polymers within the larval gut environment. These microbial shifts point to a functional adaptation of the gut microbiota in response to synthetic polymer exposure, offering valuable insights into the biodegradation processes within insect gut systems. Although we observed consistency in the presence of bacterial groups previously associated with plastic degradation, the specific roles of these microbes within the RPW gut remain unconfirmed. Specifically, while previous studies have linked *Lactococcus* to polystyrene degradation and *Pseudomonas* and *Erysipelothrix* to polyurethane breakdown, their direct contribution within RPW larvae remains unverified. Controlled experiments using sterile RPW larvae inoculated with these bacterial isolates, as well as degradation assays in culture conditions, will be crucial for confirming their role in plastic metabolism. Future metagenomic and transcriptomic analysis would help in further identification of the genes and enzymes involved, providing insights into microbial interactions and potential applications for bioremediation. RPW larvae mechanically fragmented the plastic into smaller pieces. Another possible pathway for subsequent degradation of these plastic fragments may involve oxidative enzymes such as oxidases and peroxidases produced by associated microorganisms in the gut of RPW larvae, which facilitate the breakdown of oxidized polymer fragments, However, this hypothesis requires further investigation to be confirmed.

Interestingly, RPW larvae’s ability to tolerate and degrade synthetic polymers may stem from their natural adaptation to lignin-rich palm tissues. Lignin is a complex, aromatic polymer that shares structural features with plastics like polystyrene and polyurethane, including aromatic rings and chemical recalcitrance [46,47]. This suggests that RPWs may possess enzymatic systems (e.g., laccases, peroxidases) and gut microbial communities capable of acting on similar synthetic substrates [48]. This ecological background may explain the microbial flexibility observed in plastic-fed larvae.

These results suggest significant ecological implications of plastic exposure in the gut microbiota of RPW larvae, with potential consequences for the degradation of synthetic materials. The identification of bacterial taxa associated with plastic degradation, particularly among Firmicutes and Proteobacteria, provides a promising foundation for future studies aiming to leverage gut microbiota for sustainable bioremediation applications.

## 5. Conclusions

This study evaluated the growth performance, plastic biodegradation potential, and gut microbiome of RPW larvae reared on different plastic treatments (no plastic, polystyrene, and polyurethane) under controlled conditions. The type of plastic significantly affected larvae survival and growth. Polystyrene supported a bigger head size and weight growth. By day 35, body weight and head size were similar across treatments, but survival was higher in the polystyrene and polyurethane groups than in the control group. The larvae consumed and partially degraded both plastic types without adverse effects on growth and survival, presenting microplastic fragments in their digestive systems. Polystyrene was ingested more than polyurethane. FTIR analyses showed evidence of depolymerization and oxidative changes, confirming biodegradation. The gut microbiome analysis showed bacterial taxa potentially involved in plastic digestion. The findings position RPW larvae as a promising model for plastic waste biodegradation. Further research would optimize efficiency, identify mechanisms, and address potential health risks from microplastics, paving the way for scalable, sustainable waste management solutions.

## Figures and Tables

**Table 1:** Growth performance, measured by head width (mm) and live weight (g), of RPWs fed with different plastic-containing diets.

Parameter	Treatments			*p*-Value
	Control	Polystyrene	Polyurethane	
**Head diameter** (**mm**)				
Day 20	4.10 ± 0.53 ^b^	4.40 ± 0.52 ^b^	4.40 ± 0.32 ^c^	0.153
Day 27	5.80 ± 0.63 ^a^	6.30 ± 0.67 ^a^	5.80 ± 0.42 ^b^	0.108
Day 35	6.30 ± 0.48 ^a^	6.40 ± 0.70 ^a^	6.80 ± 0.42 ^a^	0.116
*p*-value	0.000	0.000	0.000	-
**Live weight** (**g**)				
Day 20	3.61 ± 0.56	3.96 ± 0.65	4.06 ± 0.60	0.224
Day 27	3.50 ± 0.36	3.77 ± 0.61	3.83 ± 0.52	0.315
Day 35	3.35 ± 0.28	3.74 ± 0.39	3.75 ± 0.55	0.066
*p*-value	0.387	0.639	0.442	-
**Survival rate** (**%**)				
Day 20	100 ± 0.00 ^a^	100 ± 0.00	100 ± 0.00	-
Day 27	78.89 ± 11.70 ^b^	100 ± 0.00	100 ± 0.00	0.013
Day 35	41.11 ± 6.94 ^c^	100 ± 0.00	100 ± 0.00	0.000
*p*-value	0.000	-	-	

The data were analyzed using an analysis of variance (ANOVA) test, with the results expressed as mean values ± standard deviations. Means within the same column that have different superscripts are considered significantly different at the *p* < 0.05 level.

## Data Availability

The original contributions presented in this study are included in the article. Further inquiries can be directed to the corresponding authors.

## Abbreviations

The following abbreviations are used in this manuscript:

FTIR	Fourier Transform Infrared Spectroscopy
PDB	Potato dextrose broth
PS	Polystyrene
PU	Polyurethane
RPW	Red palm weevil
YPDB	Yeast-enriched potato dextrose broth
